# Catalysts of change: Japan’s universities for international research excellence

**DOI:** 10.1038/s44319-025-00467-9

**Published:** 2025-05-12

**Authors:** Noriko Osumi

**Affiliations:** Tohoku University Graduate School of Medicine, Sendai, Japan

**Keywords:** Economics, Law & Politics, Science Policy & Publishing

## Abstract

The designation of Tohoku University as Japan’s first University for International Research Excellence may mark a starting point for the overdue reform of Japan’s higher-education system to become more international and competitive.

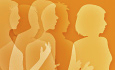

In November 2024, Tohoku University was named Japan’s first University for International Research Excellence (UIRE) under a new government initiative (MEXT, 2022) aimed at transforming Japan’s global competitiveness in academic research. This designation, backed by a ¥10 trillion national endowment fund, recognizes institutions with visionary strategies for research, cultivating talent, and societal impact. For Tohoku University, this marks not only a major achievement, but also a commitment to shaping the future of Japanese academia in a rapidly changing global landscape and to serve as a blueprint and inspiration for other universities.

Higher education with an international perspective began in Japan during the Meiji era when the country opened to Western influences. After World War 2, the university system was further expanded and developed, and a significant number of new universities were established in short time. Unfortunately, and despite its solid higher-education system, the international standing of Japanese universities has slowly declined during the past 30 years, largely owing to a lack of modernization and internationalization. The establishment of the “National University Corporation” framework in April 2004 marked a significant reform in Japanese higher education. It was designed to grant national universities greater autonomy from the government while maintaining public accountability, with the goals of improving management efficiency and enhancing international competitiveness. However, even two decades later, most ‘national’ universities remain heavily dependent on public subsidies. Only few have developed alternative funding models, such as private donations or endowments, making financial independence a continuing challenge.

Researchers in Japan also face numerous challenges. Since the incorporation of national universities, operational governmental funding has been reduced which has led to an overall decline in research funding for most scientists. At the same time, new regulations—such as stricter controls over toxic substances or security export procedures—have significantly increased administrative burdens.

Moreover, the number of young Japanese researchers going abroad has been declining, partly owing to an increase in the number of domestic postdoctoral positions. The low number of foreign researchers working in Japan is another sign that internationalization is stagnating (NISTEP, 2023). Given Japan’s declining birthrate, attracting students from abroad has become vital for the sustainability of universities and research generally. Yet, major obstacles persist. There is a shortage of faculty who are capable of teaching in English, and university administrative systems remain ill-equipped to provide adequate bilingual support.

The UIRE initiative, launched by Japan’s Ministry of Education, Culture, Sports, Science and Technology (MEXT) in 2023, aims to address these persistent issues and elevate selected Japanese universities to world-class research and teaching standards. This program will support the development of cutting-edge research environments, the recruitment of top-tier foreign scientists and the application of the result of research to addresses societal challenges. Through this framework, the initiative encourages universities to implement ambitious “Research Systems Strengthening Plans” and transform into globally competitive research hubs.

UIRE is financed by a ‘University Fund’ managed by the Japan Science and Technology Agency (JST) as an endowment. The grants are matched to external funding secured by the universities themselves—such as donations or joint research funding with industry. This mechanism represents a structural reform of Japan’s national university system. Traditionally, researchers in Japanese academia have shown limited engagement with practical application of their work or its social impact. However, the UIRE initiative is expected to change this mindset to encourage universities to become more outward-facing and socially engaged. Apart from supporting more applied research, this reform is also meant to address a long-standing issue in Japanese higher education: the need to build dynamic, globally attractive environments that can recruit and retain top international talent (Yonezawa, 2023a, 2023b, 2024).

The call for applications for the UIRE started in December 2022, and 10 universities submitted their draft plans in March 2023. Interviews were conducted by the Advisory Board in June followed by site visits at Tohoku University, the University of Tokyo, and Kyoto University. When Tohoku University was selected as the sole candidate for accreditation in September 2023, everybody wondered “Why Tohoku, not Tokyo or Kyoto?”

In fact, Tohoku University is neither the number one in terms of the number of scientists, or amount of research grants, nor in terms of number and impact of scientific publication. The reason why it was awarded the UIRE status in 2024 was because its plan, structured around three core principles Impact, Talent, and Change, included the most radical measures. “Commitment for Impact” means creation of social value based on research excellence and innovation. “Commitment for Talent” includes recruitment of talented people with diverse background from abroad. “Commitment for Change” involves readiness for reforms and responsive governance. There are 19 detailed strategies to achieve these goals—the following lists some examples.

Early and even mid-career researchers at the position of assistant professors often still work under the supervision of full professors in the same laboratories. This traditional system has been reformed to establish ‘research units’, where full professors, associate professors and assistant professors each conduct their own, independent research. This strategy will increase the number of research units from approximately 800 to about 1800. Moreover, a research environment in which early-career researchers can play a more active role is currently being developed through the enhancement of core facilities, including an increase in technical staff, and the recruitment of university research administrators.

Research support is essential to ensure the independence of early-career researchers. Tohoku University will use the UIRE funding to increase the number of support staff by more than 1000. This includes staff at core facilities, as well as research administrators (URAs) to provide support for funding applications and industry-academia collaboration.

The plan by Tohoku University also sets specific targets to be achieved within the next 25 years, such as the number and ratio of “top 10%” publications, income from intellectual property rights, ratio of international students, researchers and executive members, the ratio of female researchers, as well as self-generated income.

To facilitate interdisciplinary collaboration, Tohoku University is expanding its institutional support systems, including the Human Resources Management Office, the URA Center and the Core Facilities Center. Additionally, to strengthen partnerships with industry and startups, the Head Office of Enterprise Partnerships supports individuals with diverse talents and backgrounds to foster an environment of inclusive innovation.

Tohoku University has also launched a new framework to drive institutional transformation. As part of this initiative, the university is developing a 40,000 m² Science Park on its Aobayama Campus as a hub for research and innovation. The next-generation 3 GeV synchrotron radiation facility, known as “Nano Terasu,” is already in use by stakeholders from various sectors through a coalition-based system. The overarching goal is to bridge academic research with industrial R&D, fostering collaboration among researchers and companies from diverse backgrounds. This represents a significant shift in Japanese academia from traditionally inward-looking structures to globally connected innovation ecosystems that support advanced technological development.　Specifically, the university has established a comprehensive approach that centers on three key pillars: Open Innovation Strategy Organization, which plays a pivotal role in launching large-scale collaborative research projects with industry, government and end users; Startup Support and Entrepreneurial Development, exemplified by the Tohoku University Startup Garage; and the development of Co-Creation Hubs and regional collaboration with Sendai City and Miyagi Prefecture to foster deep-tech startup creation and regional innovation.

Tohoku’s strategy also rethinks traditional academic hierarchies. A flatter and more flexible organizational model will support early-career researchers through its own funding, interdisciplinary units and international collaborations. Unusually for a Japanese university, Tohoku has introduced a partner support system, to support research partners to join and participate in the community. Marie-Pierre Favre, who served as Vice President for International Relations at INSA Lyon for 10 years, has arrived as Chief Global Officer in April 2025. She is proceeding with the comprehensive internationalization of the university covering education, research, recruitment, and promotion of diversity. In addition, a major recruitment drive has been initiated to invite world-renowned researchers and to recruit research support staff on a large scale.

Importantly, the university’s vision extends beyond scientific advancement. Its leadership places strong emphasis on connectivity and creativity as core values. Success is not measured solely by the number of publications or their impact factors. As such, there is a growing need to adopt new indicators of achievement. One example is the university’s support for research-based startups, encouraging the commercialization of academic discoveries and fostering innovation with real-world impact. It is about building a research community that based on diversity, international partnerships and social contributions. In the words of current President Teiji Tominaga, who took over the baton from former President Hideo Ohno, Tohoku University aims to “move and shake” academia.

As the first UIRE institution, Tohoku University now bears the responsibility of being a national benchmark. Its progress will be closely watched, both by domestic policymakers and international stakeholders. If successful, it could trigger a broader transformation across Japan’s higher-education landscape from fragmented reforms towards a coherent, integrated strategy. As an academic who has witnessed Tohoku University’s evolution over decades, I believe that the UIRE accreditation is not a final destination but the beginning of a bold and necessary reform of the whole Japanese university system: one that embraces openness, excellence and international leadership in science.

## Supplementary information

Peer Review File
