# Supplementary material for: Catalysts of change: Japan’s universities for international research excellence
Source: EMBO Rep. 2025 May 12;26(11):2797–9. doi: 10.1038/s44319-025-00467-9 (PMC12152159; doi:10.1038/s44319-025-00467-9)
Supplement: Supplementary file 1 — Peer Review File [file 44319_2025_467_MOESM1_ESM.pdf]

EMBO reports

Catalysts of Change: Japan's Universities and International Research Excellence

Noriko Osumi

Corresponding author(s): Noriko Osumi (noriko.osumi.c7@tohoku.ac.jp)

Review Timeline:

Submission Date:	28th Apr 25
Accepted:	28th Apr 25

Editor: Holger Breithaupt

Transaction Report: This article type is not peer reviewed.
